# Quality of life and tolerability of B-cell directed therapy of multiple sclerosis with ofatumumab in a patient-centered real-world observational study

**DOI:** 10.1007/s00415-024-12581-0

**Published:** 2024-07-22

**Authors:** Anna-Sophia Karl, Rafael Klimas, Melina Katsimpoura, Melissa Sgodzai, Simon Theile-Ochel, Philip Lennart Poser, Barbara Gisevius, Simon Faissner, Anke Salmen, Ilias Nastos, Ralf Gold, Jeremias Motte

**Affiliations:** 1 Clinic for Neurology, St. Josef Hospital, Ruhr University Bochum, Gudrunstraße 56, 44791 Bochum, Germany; 2 Specialist Practice for Neurology, Bochum, Germany

**Keywords:** Ofatumumab, B-cell depletion, Multiple sclerosis, Anti-CD20 monoclonal antibody

## Abstract

**Introduction:**

Ofatumumab (Kesimpta^®^) is a subcutaneous CD20-targeting antibody approved in Germany in 2021 for the treatment of relapsing multiple sclerosis (RMS). After careful instruction, patients can administer the treatment themselves. We previously reported data of 101 patients (Klimas et al. in Nervenarzt 94:923–933, 2023). The objective of this longitudinal study is to explore the tolerability and acceptability of ofatumumab from a patient perspective over a follow up period of 6 months.

**Methods:**

In this prospective observational real-world study, we report follow up data of 81 patients. We evaluated sociodemographic data, disease duration, duration and side effects of ofatumumab use, expanded disability status scale (EDSS), Beck Depression Inventory II (BDI-II), Short-Form 36 (SF-36), Fatigue Scale of Motor and Cognitive Functions (FSMC), and modified Multiple Sclerosis Functional Composite Test (MSFC). In addition, we asked for subjective treatment outcomes, such as impact on quality of life, walking distance, concentration, mood, medication adherence, fatigue and the subjective course of MS on a numerical rating scale (1 = very negative; 5 = very positive). Furthermore, treatment discontinuations were recorded.

**Results:**

The average duration of ofatumumab treatment was 10 months. In comparison to previous published data of our cohort, patients reported a significant increase in headache (10% up to 26%, p = 0.004) and limb pain (5% up to 26%, p < 0.001) as persistent side effects after the injections. More patients reported a very positive effect (p < 0.0001) on quality of life. 4 confirmed relapses occurred but no EDSS worsening, and no treatment discontinuations were documented during the observation period.

**Discussion:**

As previously described, our prospective study indicates that patients have a good tolerability of ofatumumab, precisely because of the mild and few side effects at the first administration. However, the longer the observation period, the more headaches and limb pain occurred after the injections. Despite this, patients’ subjective quality of life improved. There were no discontinuations during the follow-up period, with the limitation of a high loss to follow-up.

**Supplementary Information:**

The online version contains supplementary material available at 10.1007/s00415-024-12581-0.

## Introduction

Multiple sclerosis (MS) is a chronic autoimmune disorder characterized by inflammation, demyelination, and neurodegeneration within the central nervous system. The disease can present in various forms, with relapsing–remitting MS (RRMS) being the most common, followed by secondary progressive MS (SPMS) which can exhibit active relapsing symptoms contributing to disability accumulation. Early initiation of disease-modifying therapies (DMTs) has been shown to reduce the risk of disability progression, underscoring the importance of effective and well-tolerated treatment options [1–4].

B-cell depleting therapies, particularly monoclonal antibodies (MoAbs) targeting the CD20 antigen on B cells, have emerged as a pivotal advancement in the treatment landscape for MS [5–7]. Ofatumumab (Kesimpta^®^), a fully human anti-CD20 antibody, was approved in Germany in 2021 for the treatment of RMS. Unlike its predecessor ocrelizumab, ofatumumab is administered subcutaneously, allowing patients the convenience of self-administration after appropriate training. Clinical trials, notably the ASCLEPIOS I and II, demonstrated the superior efficacy of ofatumumab compared to teriflunomide, highlighting its potential in delaying disability progression, reducing relapse rates, and diminishing central nervous system inflammation [8–11].

Despite these promising clinical trial results, real-world data on the tolerability and impact of ofatumumab on patient-reported outcomes in everyday clinical practice remain limited. Our initial study demonstrated that ofatumumab administered subcutaneously was highly accepted and well-tolerated [12]. Here, our study aims to bridge the gap between real-word data and clinical trials by providing observational data from a patient-centered perspective over a 6-month follow-up period. This study evaluates the tolerability, safety, and subjective treatment outcomes of ofatumumab in a real-world setting, focusing on quality of life, functional status, and side effect profile.

Our specific research questions include:

1. How do reported side effects evolve over the course of treatment?
2. How does ofatumumab influence quality of life and functional outcomes during the observation period?
3. Are patient-reported outcomes and Multiple Sclerosis Functional Composite Test (MSFC) scores stable or improved during the treatment period?
4. Do patients report relapses under therapy with ofatumumab?

This prospective observational study aims to provide comprehensive insights into the real-world application of ofatumumab, supporting its role as a viable treatment option for patients with relapsing MS.

## Methods

### Patients and study design

**Fig. 1:**
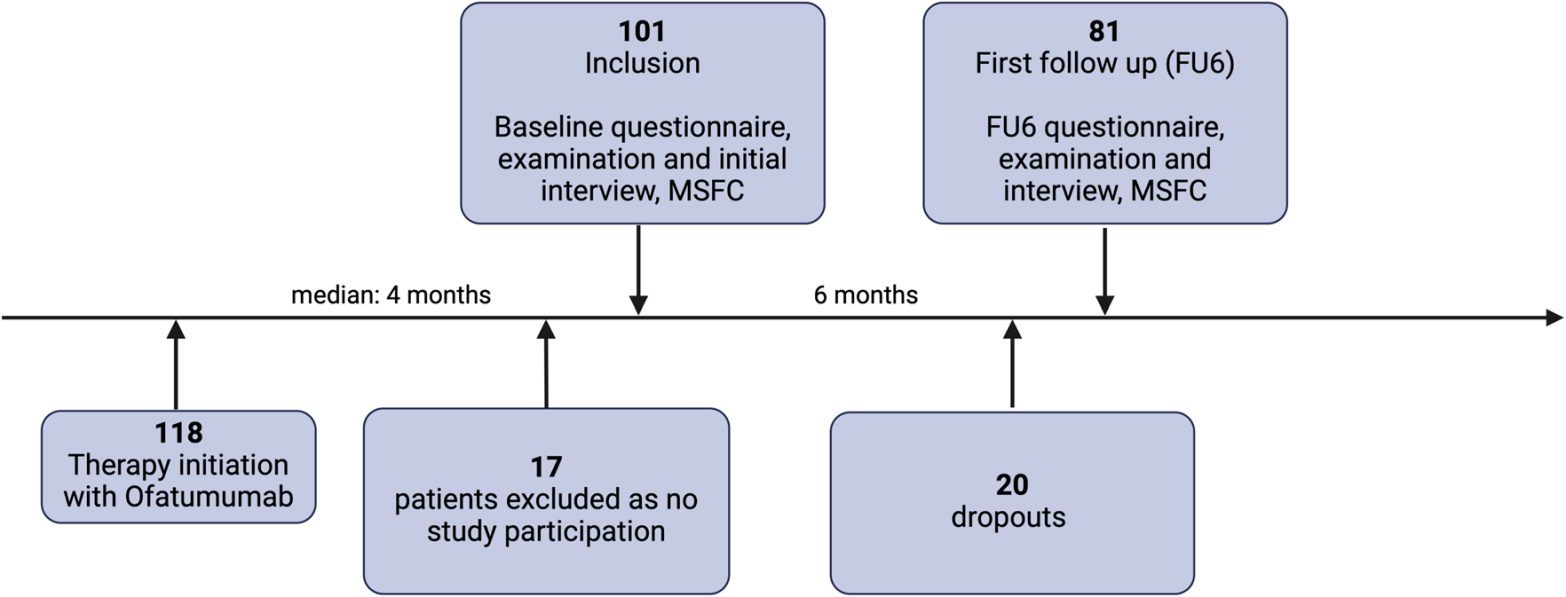
Study design. 118 patients were enrolled in the study after medical adjustment. 17 patients did not want participate in the study, leaving 101 patients in the study. At baseline, patients were required to complete a questionnaire and provide their disease and current medical history. Furthermore, a physical examination and MSFC were conducted. Following baseline assessments, 20 of the 101 patients either withdraw from the study for the personal reasons or could not be contacted. Subsequently, a second appointment was scheduled after a period of 6 months. 81 patients have already completed this appointment

Adult patients (male/female) who have initiated treatment with ofatumumab as indicated during routine clinical treatment decision-making qualified for the study and have been asked to participate after treatment initiation at the Department of Neurology, St. Josef University Hospital Bochum, and in a large MS specialist practice in the Ruhr region (Dr. med. Nastos) between September 2021 and November 2023. No additional in-/exclusion criteria were set. After enrollment, clinical data and scores were collected at baseline [12], and a follow-up appointment was scheduled for 6 months later (Follow-up 6 months (FU1)) (Fig. 1).

### Standard protocol approval, registrations, and patient consent

The ethics committee of the Medical Faculty of Ruhr-University Bochum, Germany approved our study (reg.-no. 20-6827). Written informed consent was obtained from all patients. All procedures performed in studies involving human participants were in accordance with the ethical standard of the institutional and/or national research committee and with the 1975 Helsinki Declaration and its later amendment or comparable ethical standards.

### Outcome analyses

The systematic data collection included the following elements for each of the two time points:

- **Sociodemographic data:**
  - age and sex
- **Disease-specific data:**
  - disease duration
  - EDSS
  - relapses during treatment
- **Ofatumumab treatment data:**
  - duration of treatment with ofatumumab
  - treatment discontinuations
  - side effects
- **Patient reported outcomes (PROs):**
  - Beck depression inventory II (BDI-II)
  - Short form 36 (SF-36)
  - Fatigue Scale for Motor and Cognitive Functions (FSMC)
- **Functional tests:**
  - modified Multiple Sclerosis Functional Composite Test (MSFC)
  - Times 25-Foot Walk Test (T25FT)
  - 9 Hole Peg Test (NHPT)

At FU1, questionnaires and documentation collected during the study visit were used to collect data. At the first visit, we assessed early side effects (within 48 h after the first injection) and late side effects (during one week after the first injection) as described previously. We here assessed persistent side effects from baseline to FU1, regardless of the time of injection. Additionally, we recorded participants’ subjective experiences of handling the self-administration, as well as treatment impact on quality of life, walking distance, concentration, mood, medication adherence, fatigue and the subjective course of MS on a numerical rating scale (1 = very negative/hard/inaccurate, 2 = negative/hard/inaccurate, 3 = neutral, 4 = positive/ease/accurate, 5 = very positive/easy/accurate, 0 = “I don’t know”). In case of treatment discontinuation, reasons were explored. For unanswered FSMC and BDI questions, the mean of other responses is imputed. The SF-36 questionnaire results are condensed into a physical and mental sum scale, ranging from 0 (very low quality of life) to 100 (very high quality of life), and benchmarked against a German norm population [13]. A modified MSFC was applied, omitting the PASAT due to low patient tolerance.

### Statistics

Statistical analysis was performed using SPSS (Statistical Package for the Social Sciences, version 27), GraphPad Prism (GraphPad Software, www.graph pad.com, version 9.5.0) and SankeyMATIC. Parametric data are presented as mean with standard deviation (SD), and nonparametric data as median with interquartile range (IQR) if not stated otherwise. Nominal and dichotomous variables are presented as counts and percentages. Clinical characteristics were compared between groups using Wilcoxon matched-pairs signed rank test for numerical non-normally distributed paired values, chi-squared test (χ^2^-test) for nominal variables and McNemar test (*χ*^2^-test) for dichotomous variables. The statistically significant threshold was set at p-value < 0.05. For multiple testing, the p-value was corrected according to Bonferroni. The primary outcome is defined as improvement in the application questions. Secondary outcomes are stability of EDSS, PROs and MSFC.

## Results

### Patient characteristics

Between September 2021 and November 2023, 101 patients newly treated with ofatumumab were recruited. Follow-up questionnaires and study visits were performed on 81 patients at FU1. 20 of the 101 patients (20%) either withdrew from the study for personal reasons or could not be contacted. At follow-up time point 67 patients (83%) were female, and the mean age was 45 years (SD: 10, range 23–70). The median length of time since initial diagnosis was 7.5 years (IQR: 10.5, range 0–35). On average, patients were treated with ofatumumab for 10 months (IQR: 5.25, range 4–19, n = 76, Table 1). The pre-treatments and EDSS scores at baseline of the patients enrolled in the study can be found in the preliminary work [12]. The median FU1 EDSS score remained stable at 2 (IQR: 1.5, range 1.0–6.5, n = 76) after 6 months observational period (Table 1).

**Table 1:** Patients characteristics at FU1

Patients, n	81
Female patients, n (%)	67 (83)
Age of patients (years), mean (SD), n	45 (10), 81
Disease duration since diagnosis, years median (IQR), n	7.5 (10.5), 10
Disease duration since manifestation, years median (IQR), n	9 (11), 79
Month since first ofatumumab injection, median (IQR), n	10 (5.25), 76
EDSS, median (IQR), n	2.0 (1.5), 76

### Four confirmed relapses and no treatment discontinuation in the 6-month observational period

5 patients (6%) reported a relapse event during the observation period. One was associated with respiratory infection, leading to a confirmed relapse event in 4 patients (5%). At the time of the relapse, the mean disease duration was 94 ± 63 months, Ofatumumab was taken by the patients for an average of 13 ± 2 months, and the mean EDSS was 3.0. Each relapse had up to 6 different symptoms. Gait disturbance and gait unsteadiness were the most common relapse symptoms, accounting for 3/4 of cases. This was followed by sensory disturbance affecting more than one part of the body (2/4 of cases), paralysis or weakness of more than one part of the body, e.g., hemiplegia (2/4 of cases) and sexual disorders (2/4 of cases). Each relapse was treated with intravenous corticosteroids and recovered fully. During the 6-month observation period, none of the 81 patients discontinued treatment. Details are displayed in supplementary Fig. 1.

### Increasing severity of side effects with predominantly headache and limb pain

To enable a longitudinal comparison of side effects, we analyzed the 81 patients intraindividually. We used the McNemar test to compare early (within 48 h after the first injection) and late (within 1 week after the first injection) side effects at baseline with FU1 (Table 2). When comparing the early side effects reported at baseline to those reported after 6 months, a significantly higher number of individuals reported the absence of those side effects (p < 0.001). Additionally, side effects such as chills and fever (p < 0.001), headache (p = 0.004), and limb pain (p < 0.001) were reported significantly less frequently. However, it is important to note that certain side effects, such as headache (p = 0.004) and limb pain (p = < 0.001), increased significantly when compared to the late side effects. In conclusion, it can be stated that the incidence of long-lasting side effects has increased, while that of short-lasting side effects has decreased.

**Table 2:** Side effects of longitudinal cases

	A: early side effects	B: late side effects	C: FU1 side effects	A vs. C	B vs. C
Total n	81	81	81		
No side effects, n (% of cases)	21 (26)	54 (67)	43 (53)	< 0.001	0.043
Chills/fever, n (% of cases)	36 (44)	0 (0)	4 (5)	< 0.001	–
Headache, n (% of cases)	37 (46)	8 (10)	21 (26)	0.004	0.004
Pain in the limbs, n (% of cases)	41 (51)	4 (5)	21 (26)	< 0.001	< 0.001
Respiratory difficulties, n (% of cases)	2 (3)	1 (1)	7 (9)	0.125	–
Skin rash, n (% of cases)	1 (1)	1 (1)	1 (1)	1	1
Urinary tract infection, n (% of cases)	1 (1)	0 (0)	6 (7)	0.125	–
Local inflammation at the injection site, n (% of cases)	0 (0)	0 (0)	1 (1)	–	–
Other, n (% of cases)	17 (21)	6 (7)	14 (17)	0.648	

Early (within 48 h after the first injection), late (during 1 week after the first injection) and side effects at follow-up after 6 months (FU1). McNemar test between baseline early side effects and FU1 and baseline late side effects and FU1

**Fig. 2:**
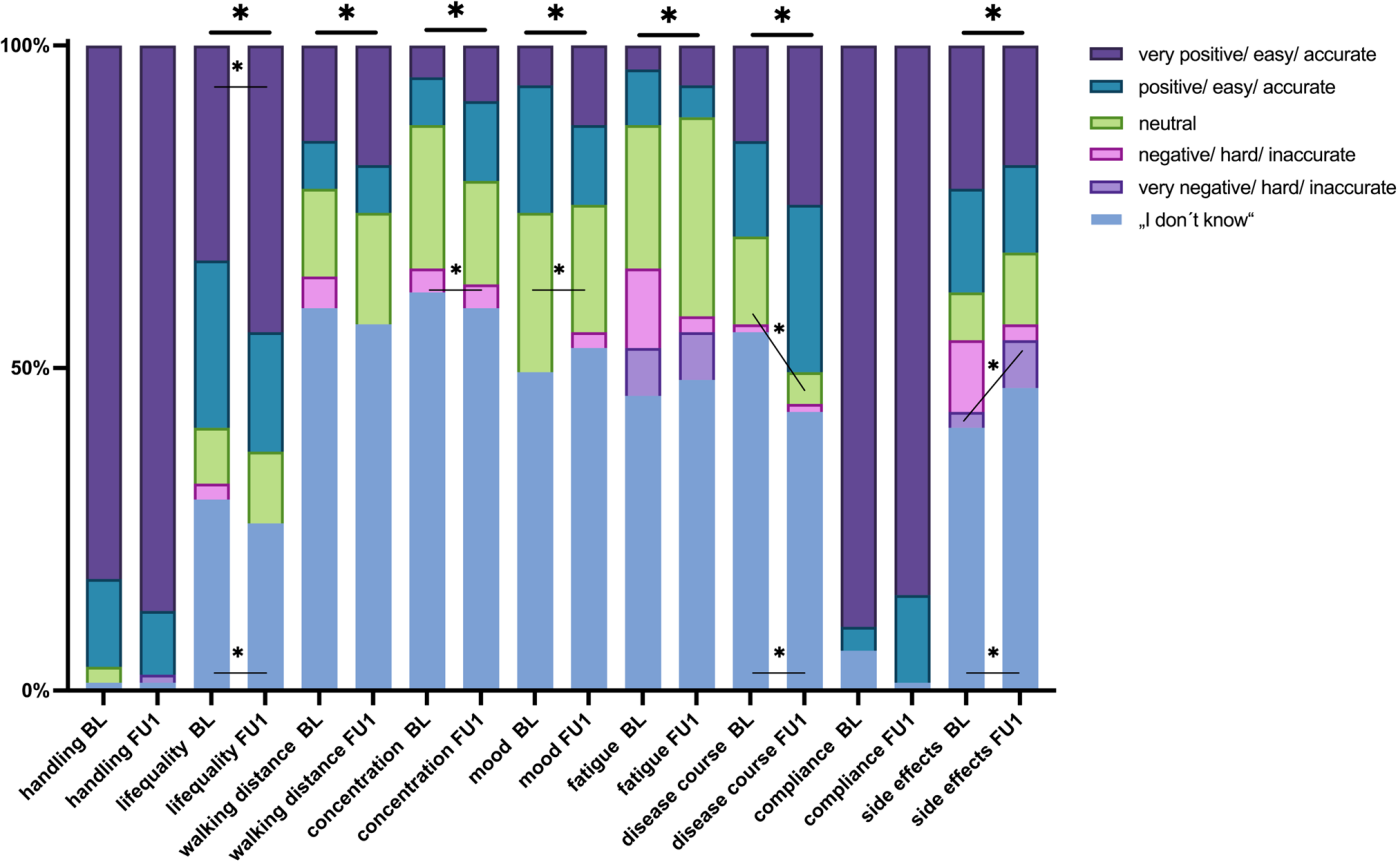
Application questions. Impact of ofatumumab on various patient-Reported outcomes over time. Figure illustrates the changes in the patient-reported outcomes over the course of ofatumumab treatment during a 6 month observation period. Parameters accessed include quality of life, walking distance, concentration, mood, medication adherence, and fatigue from a patients subjective perspective. Statistically significant differences were determined using the chi-squared test. Drug-handling: p = 0.099. Quality of life: p = 0.002. Walking distance p < 0.001. Concentration: p < 0.001. Mood: p = 0.010. Fatigue: p < 0.001. Disease course: p < 0.001. Compliance: p = 0.085. Ofatumumab side effects: p < 0.001. The symbols within the bars indicate significance after post-hoc testing with Bonferroni correction

The severity of side effects at FU6 was perceived by 19% of the participants (n = 15) as “very mild”, 14% (n = 11) “mild”, 11% (n = 9) “neutral”, 2% (n = 2) “severe”, 7% (n = 6) “very severe” and 47% (n = 38) did not know how to answer. The results of the post-hoc test, which was conducted using the Bonferroni correction, indicated that the observed significance was since a greater proportion of patients selected the answers “very severe” and “I don’t know” than expected (p < 0.0001, Fig. 2).

### High patient acceptance and positive impact on quality of life

We previously reported the baseline data in a separate paper [12]. The FU1 data can be found in Fig. 2.

88% (n = 71) of the patients reported finding the medication handling “very easy” at follow-up, 10% (n = 8) “easy”, 1% (n = 1) “very hard” and one patient (1%, n = 1) selected the “I don’t know” option. There was no statistical difference between baseline and FU1 (p = 0.099).

The influence of ofatumumab on quality of life was rated as “very positive” by 44% (n = 36) and “positive” by 19% (n = 15) of participants. 11% of participants (n = 9) selected “neutral” and 26% (n = 21) were unsure how to respond. Here, a significant difference in response options (p = 0.002) was observed. Following Bonferroni correction, more patients reported a “very positive” effect (p = 0.0001), and significantly fewer patients selected the “I don’t know” option (p = 0.001) than expected.

19% (n = 15) of participants rated the impact of ofatumumab on their walking distance as “very positive,” with another 7% (n = 6) rating it as “positive”. No changes (“neutral”) in walking distance were reported by 17% of the participants (n = 14) while 57% (n = 46) were unsure about the effect of ofatumumab. A significant difference is evident here (p < 0.001). Following Bonferroni correction, less patients reported a “very positive” effect (p = 0.0001), and significantly more patients selected “neutral” as their answer option (p = 0.0004).

The effect of ofatumumab on concentration is widely regarded as "neutral” (16% (n = 13).

9% (n = 7) experienced a very positive effect, 12% (n = 10) experienced a positive effect, 4% (n = 3) experienced a negative effect and 59% (n = 48) did not know. A statistically significant difference was found between the baseline and FU1 (p < 0.001). The Bonferroni correction indicates that a significantly higher number of patients selected “negative” compared to what was expected.

At FU1, 12% (n = 10) reported a “very positive” impact on their mood, 12% (n = 10) a positive, 20% (n = 16) neutral, and 2% (n = 2) negative. 53% (n = 43) chose “I don’t know.” The statistical significance of the results is apparent (p = 0.010) as significantly more patients selected “neutral” than expected.

Fatigue during therapy with ofatumumab was rated as “neutral” by 31% (n = 25). 2% (n = 2) reported “more fatigue” and 7% (n = 6) “much more fatigue”. “Much less fatigue” was reported by 6% (n = 5) and “less fatigue” by 5% (n = 4). 48% (n = 39) did not know. However, due to the small sample size the Bonferroni post-hoc test is unable to determine between which pairwise comparisons this significant difference exists.

85% (n = 69) of patients adhered “very accurate” to monthly self-injection. 14% (n = 1^) adhered “accurate” to monthly intervals. 1% (n = 1) chose the option “I don’t know”. There was no statistical difference (p = 0.085).

After 6 months, a significant difference (p < 0.001) was observed in the influence on subjective MS disease progression. 25% (n = 20) of patients reported a “very positive” influence, 26% (n = 21) a “positive” influence, 5% (n = 4) a “neutral” influence of ofatumumab on disease progression. However, one patient (1% (n = 1) reported a “negative” influence and 43% (n = 35) chose the option “I don’t know”. A significantly higher percentage of patients selected the response “I don’t know” (p < 0.0001) and a slightly higher percentage selected the response “neutral” (p < 0.0001).

### No significant changes in multiple sclerosis impact scale (MSIS-29)

The study utilized the MSIS-29 to analyze the effect of MS on physical (physical impact score) and psychological (psychological impact score) factors. The scores were plotted on a scale ranging from 0 (‘no effect of the disease’) to 100 (‘maximum effect of the disease’).

At FU1, the median physical score remained at 23.75 (IQR: 30, range 0–80, n = 81). In the psychological assessment, a median score 36.11 (IQR: 38.89, range 0–94.45, n = 81) was achieved at the FU1. No significant difference was observed between Baseline and FU1 for either score (Supp. Figure 2).

#### No significant changes in fatigue scale for motor and cognitive functions (FSMC)

The level of fatigue symptoms was evaluated using FSMC, distinguishing between global, motor, and cognitive fatigue. The cohort’s median score for global fatigue at FU1 was 63 (IQR: 32, range 20–100, n = 81), representing severe global fatigue (Supp. Figure 3A). With a median of 31 points at FU1 (IQR: 19, range 10–50, n = 81), indicating moderate cognitive fatigue (Supp. Figure 3B). The cohort’s median score for motor fatigue remained at 32 points (IQR: 16, range 10–50, n = 81). This indicated severe motor fatigue (Supp. Figure 3C). Compared to our previous reported data [12] there were no significant chances in neither one of the scores.

#### BDI-II remained stable, indicating no worsening of depressive symptoms

The frequency of depressive symptoms in our study cohort was assessed using the BDI-II. At FU1, the median score remained at 12 points (IQR: 16, range 0–33, n = 80). Median scores indicate a minimal level of depression. 38% (n = 31) did not suffer from depressive symptoms. 15% (n = 12) reported minimal depressive symptoms, 17% (n = 14) mild symptoms and 18% (n = 15) moderate symptoms. Severe depression was reported by 10% (n = 8) of the patients. No significant difference in BDI-II was detected during the observation period (Supp. Figure 4).

#### Short-Form (SF-36) Health Survey shows no alterations

The quality of life was evaluated using the SF-36 questionnaire. Our findings indicate that our cohort exhibited lower disease-related quality of life compared to the German norm population in both the physical (mean: 41.46 ± 11.88, min: 17.91, max: 62.40, n = 80) and mental (mean: 44.37 ± 11.63, min: 18.66, max: 62.68, n = 80) sum scales at FU1 (Tab. 3). However, no significant changes were observed in the physical sum scale after 6 months. Furthermore, no alterations were observed in the remaining scales.

**Table 3:** Short Form (36) Healthy Survey of longitudinal cases after 6 months (FU1)

	N	Minimum	Maximum	Mean	SD	Two-tailed p value
Physical sumscale	80	17.91	62.40	41.46	11.88	0.830
Mental sumscale	80	18.66	62.68	44.37	11.63	0.193
Physical functioning	81	0.00	100.00	69.25	29.78	0.282
Physical role function	81	0.00	100.00	54.63	42.41	0.461
Physical pain	81	0.00	100.00	61.21	30.93	0.309
General health perception	80	0.00	100.00	47.83	22.52	0.266
Vitality	81	0.00	100.00	39.32	24.34	0.206
Social role functioning	81	0.00	100.00	68.06	30.29	0.371
Emotional role functioning	81	0.00	100.00	63.79	44.78	0.140
Mental health	81	12.00	100.00	66.32	20.44	

### Modified multiple sclerosis functional composite (MSFC)

A modified MSFC was applied, omitting the PASAT due to low patient tolerance.

#### No significant changes in Timed 25-Foot Walk Test (T25FT)

At baseline, patients took an average of 5.14 ± 1.55 s (min: 3.3, max: 11.45, n = 63) to cover the distance. At the FU1, the patients required an average of 5.26 ± 1.97 s (min: 3.2, max: 15.1, n = 63). No significant differences were observed between the two time points.

#### 9-Hole Peg Test (NHPT) with no significant differences

At baseline, patients achieved a mean time of 20.81 ± 3.81 s (min: 14.9, max: 34.65, n = 63) using their dominant hand and 21.31 ± 3.31 s (min: 15.7, max: 30.7, n = 63) using their non-dominant hand. No significant differences were found compared to FU1, where patients completed the test using a mean time of 20.28 ± 4.25 s (min: 12.4, max: 32.75, n = 63) with their dominant hand and a mean time of 21.07 ± 3.75 s (min: 13.5, max: 29.1, n = 63) with their non-dominant hand.

## Discussion

Following a 6-month observation period, it can be demonstrated that ofatumumab has a good tolerability profile in patients due to its simple administration and mild side effect profile. In addition, clinical course assessment using PROs, EDSS and functional tests shows that most patients had a stable disease course under ofatumumab. Yet, during the observation period, 4 out of 81 (5%) patients experienced a relapse, from which all patients recovered after the administration of intravenous corticosteroids. None of the patients required plasmapheresis. The number of relapses is consistent with pivotal studies. In ASCLEPIOS I, 90 out of 454 (19%) treatment-naive patients suffered relapses in a 30-month observation period. Similarly, in ASCLEPIOS II, 95 out of 469 (20%) treatment-naive patients suffered relapses in a 30-month observation period [11]. However, it must be emphasized that comparing these studies is difficult, as they were conducted under strictly standardized conditions. Due to the small number of cases, an analysis of relapse events and risk factors was here not conducted [14]. Nevertheless, none of the 81 patients discontinued therapy, highlighting the high level of acceptance of the drug among both patients and treating physicians.

After a median of 10 months under treatment with ofatumumab, patients report a very positive subjective perception of their disease and a subjective improvement in their quality of life. Ofatumumab seems to have a high rate of side effects after the first injection, but these are subjectively mild and late side effects are rarely reported. We assume that side effects are particularly high after the first injection due to a rapid cytokine release. The same mechanism leads to the necessity of administration of methylprednisolone and antihistamines prior to infusion of ocrelizumab [15]. Of note, the side effect profile of the longitudinally observed patients changed surprisingly during the observation period. Headaches and aching limbs increased significantly compared to the late side effects at baseline and were mostly of minor intensity. We suspect that patients received a recurrent increase in inflammatory cytokine profile through ofatumumab, which may trigger the adverse effects. Experiments and further studies are needed to proof this hypothesis. Another explanation is that multiple injections of ofatumumab may induce an increased psychological sensitivity to new side effects, which may then be perceived as more severe. A number of studies have been conducted which examine the relationship between anxiety and self-injection [16]. However, further research is required to determine how and to what extent anxiety can manifest itself, and whether this can have an impact on attention to side effects.

As demonstrated in our previous study [12], our cohort is mildly affected with a median EDSS of 2. This EDSS stability is also evident in terms of fatigue. Our patients report high global fatigue (median: 63) and psychological burden in the MSIS-29 (median: 36.11), but do not accelerate disease progression. In addition, Timed 25-Foot Walk Test as well as 9-hole peg test and BDI show disease stability in our cohort.

While DMT is not expected to improve the course of the disease, our patients report some positive effects. As expected, our cohort had a lower disease-related quality of life compared to the normal German population in the SF-36 at BL. In the follow up evaluation, no significant alterations were observed in the SF-36. The subjective assessment of quality of life, however, revealed a “very positive” effect of ofatumumab. The discrepancy between these findings could be since the SF-36 does not assess disease-specific quality of life. Moreover, the scientific analysis of subjective experience reports is particularly challenging and therefore must be interpreted with caution. Subjective evaluations of walking distance (p < 0.001), concentration (p < 0.001), mood (p = 0.010), fatigue (p < 0.001) and disease course (p < 0.001) depicted significant improvements between baseline and follow-up. We suggest that these, positive effects are mainly due to the form of administration and the knowledge that a highly effective substance is being received. Although some patients have previously undergone highly effective therapy, the positive perception may be influenced by the ease of use and the patients’ sense of self-determination and self-efficacy.

A study conducted in Canada demonstrated that ofatumumab is significantly more cost-effective than other DMTs approved for RRMS in Canada [17]**.** The simple and accessible route of administration of ofatumumab enables a high degree of autonomy and independence from hospitalization and long stays in infusion clinics compared to other immunotherapies. Additionally, the subcutaneous administration method enhances patient convenience and adherence, thereby facilitating earlier initiation of treatment [18]**.**

Our study has some limitations. Patients were not included in the study right from the start when they initiated ofatumumab. Therefore, not all patients took the drug for the same duration, making it challenging to compare its benefits and drawbacks and making PROs more difficult to interpret. The subjective questionnaires were not validated and therefore should be interpreted with caution. Furthermore, we employed a modified shorter version of the MSFC, in which neither the PASAT nor its alternative, the SDMT, were utilized. There were 20 dropouts from baseline to follow-up. This means that relapse rates and adherence to treatment can only be assessed to a limited extent. The proportion of patients who were recorded in our structured and exploratory study, especially at follow-up, could be further increased. It is possible that a center effect exists within this cohort.

## Conclusion

Patients reacted positively to the drug, with both good tolerance and acceptance rates. However, after a median of 10 months on ofatumumab, especially late side effects are significantly more common than at baseline, when patients were on ofatumumab for a median of 4 months. Headache and limb pain have increased significantly. Nevertheless, there have been no instances of treatment discontinuation due to side effects or relapse symptoms. In our opinion, the question of long-term tolerability has not yet been conclusively resolved. Further observations are needed to demonstrate the long-term therapeutic effects of administering ofatumumab for more than one year and the mechanisms underlying adverse effects.

## Supplementary Information

Below is the link to the electronic supplementary material.

Supplementary file1 (PNG 1772 KB)

Supplementary file2 (PNG 2726 KB)

Supplementary file3 (PNG 3944 KB)

Supplementary file4 (PNG 2439 KB)

## Data Availability

The data collected for this study can be requested upon reasonable request.
