# Supplementary figures and images for: Quality of life and tolerability of B-cell directed therapy of multiple sclerosis with ofatumumab in a patient-centered real-world observational study
Source: J Neurol. 2024 Jul 22;271(9):6080–8. doi: 10.1007/s00415-024-12581-0 (PMC11377633; doi:10.1007/s00415-024-12581-0)

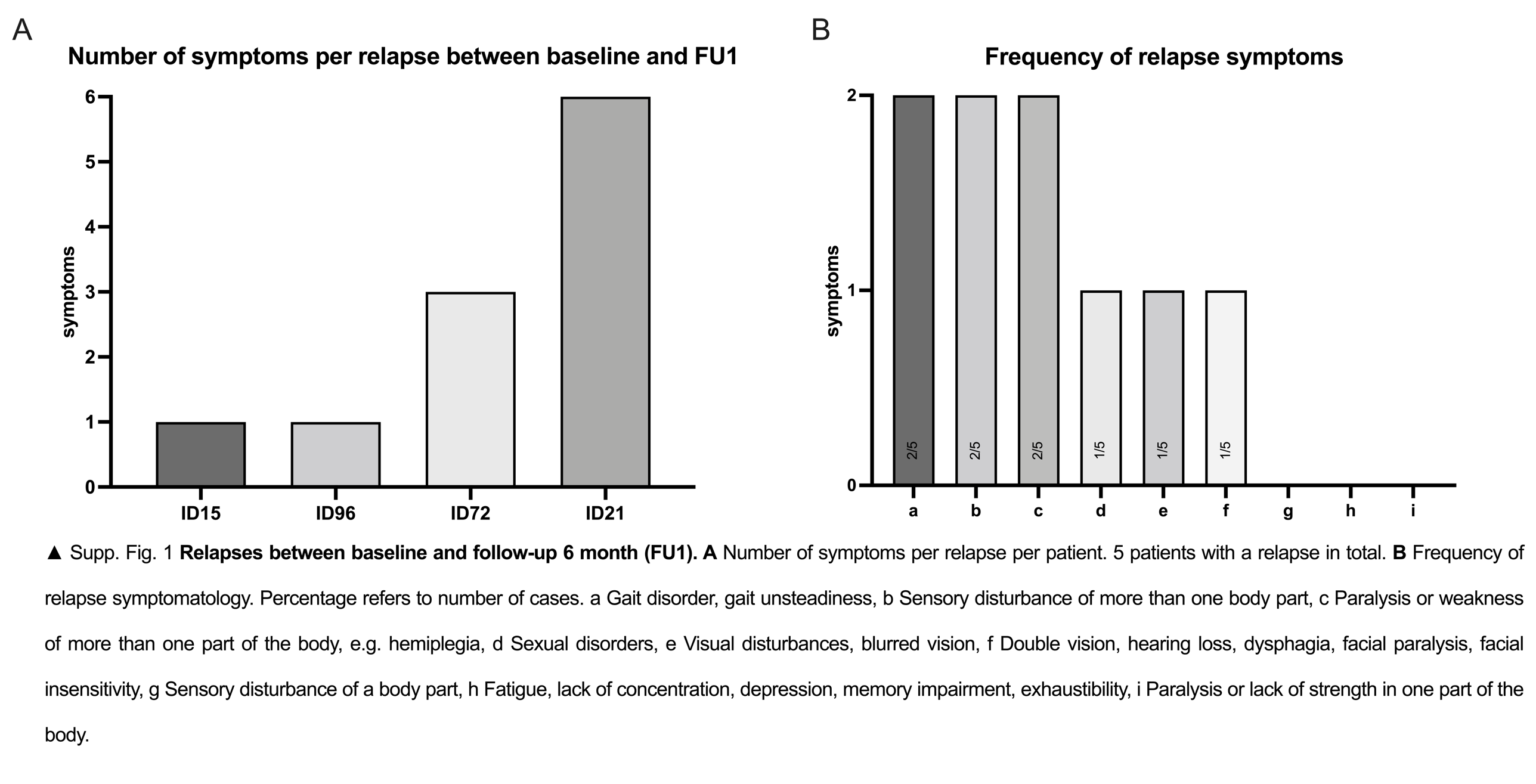

Supplement: Supplementary file 1 — Supplementary file1 (PNG 1772 KB) [file 415_2024_12581_MOESM1_ESM.png]

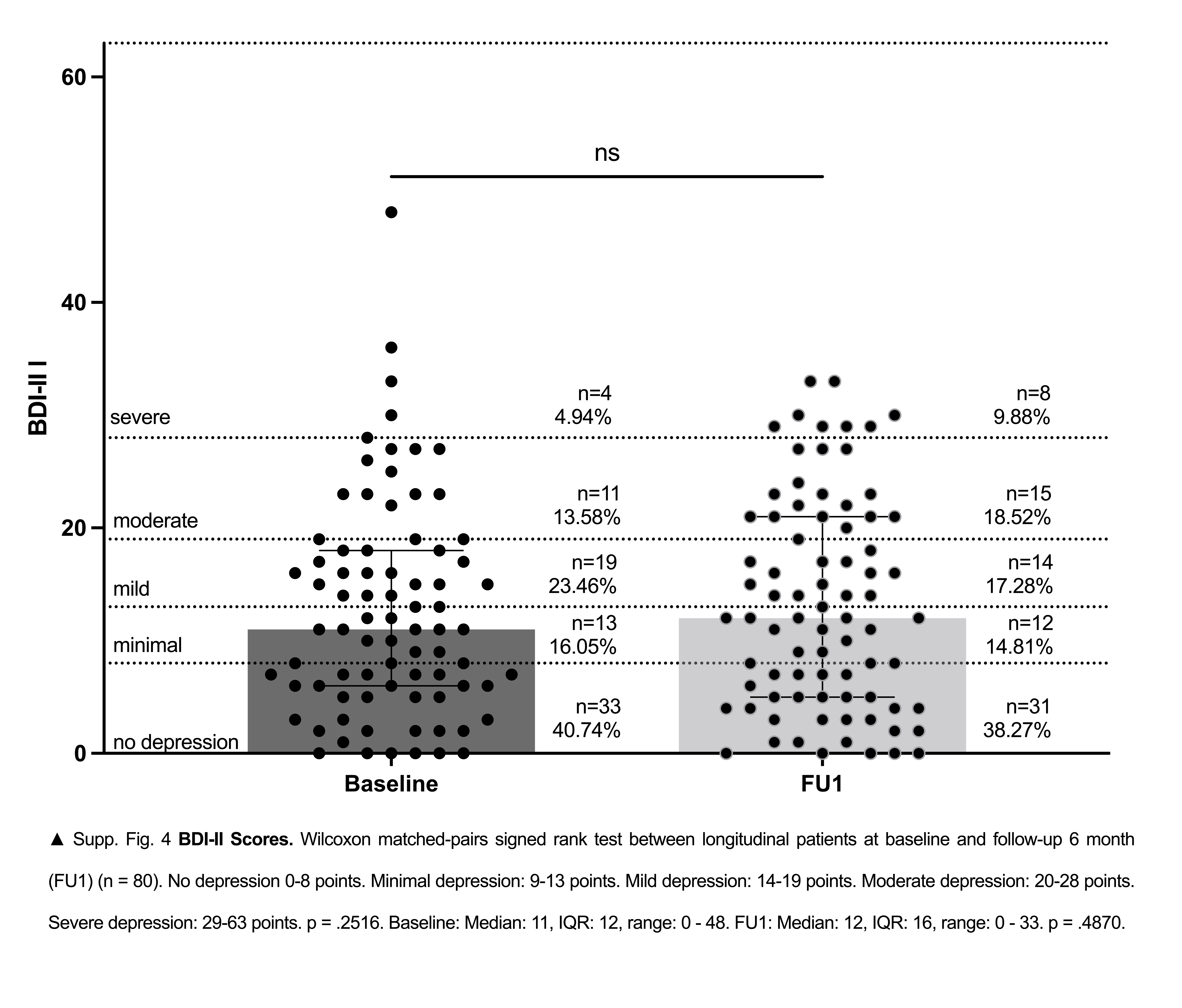

Supplement: Supplementary file 4 — Supplementary file4 (PNG 2439 KB) [file 415_2024_12581_MOESM4_ESM.png]
